# Identifying and validating MMP family members (MMP2, MMP9, MMP12, and MMP16) as therapeutic targets and biomarkers in kidney renal clear cell carcinoma (KIRC)

**DOI:** 10.32604/or.2023.042925

**Published:** 2024-03-20

**Authors:** KUNLUN LI, DANDAN LI, BARBOD HAFEZ, MOUNIR M. SALEM BEKHIT, YOUSEF A. BIN JARDAN, FARS KAED ALANAZI, EHAB I. TAHA, SAYED H. AUDA, FAIQAH RAMZAN, MUHAMMAD JAMIL

**Affiliations:** 1 The Second Affiliated Hospital of Harbin Medical University, Harbin Medical University, Harbin, China; 2 Department of Pharmaceutical Engineering, Jiangsu Ocean University, Lianyungang, China; 3 Department of Biological Engineering, University of Salford, Salford, UK; 4 Department of Pharmaceutics, College of Pharmacy, King Saud University, Riyadh, Saudi Arabia; 5 Department of Animal and Poultry Production, Faculty of Veterinary and Animal Sciences, Gomal University, Dera Ismail Khan, Pakistan; 6 Department of Arid Zone Research, PARC institute, Dera Ismail Khan, Pakistan

**Keywords:** KIRC, MMP gene family, Chemotherapy, Overall survival

## Abstract

Kidney Renal Clear Cell Carcinoma (KIRC) is a malignant tumor that carries a substantial risk of morbidity and mortality. The MMP family assumes a crucial role in tumor invasion and metastasis. This study aimed to uncover the mechanistic relevance of the MMP gene family as a therapeutic target and diagnostic biomarker in Kidney Renal Clear Cell Carcinoma (KIRC) through a comprehensive approach encompassing both computational and molecular analyses. STRING, Cytoscape, UALCAN, GEPIA, OncoDB, HPA, cBioPortal, GSEA, TIMER, ENCORI, DrugBank, targeted bisulfite sequencing (bisulfite-seq), conventional PCR, Sanger sequencing, and RT-qPCR based analyses were used in the present study to analyze MMP gene family members to accurately determine a few hub genes that can be utilized as both therapeutic targets and diagnostic biomarkers for KIRC. By performing STRING and Cytohubba analyses of the 24 MMP gene family members, MMP2 (matrix metallopeptidase 2), MMP9 (matrix metallopeptidase 9), MMP12 (matrix metallopeptidase 12), and MMP16 (matrix metallopeptidase 16) genes were denoted as hub genes having highest degree scores. After analyzing MMP2, MMP9, MMP12, and MMP16 via various TCGA databases and RT-qPCR technique across clinical samples and KIRC cell lines, interestingly, all these hub genes were found significantly overexpressed at mRNA and protein levels in KIRC samples relative to controls. The notable effect of the up-regulated MMP2, MMP9, MMP12, and MMP16 was also documented on the overall survival (OS) of the KIRC patients. Moreover, targeted bisulfite-sequencing (bisulfite-seq) analysis revealed that promoter hypomethylation pattern was associated with up-regulation of hub genes (MMP2, MMP9, MMP12, and MMP16). In addition to this, hub genes were involved in various diverse oncogenic pathways. The MMP gene family members (MMP2, MMP9, MMP12, and MMP16) may serve as therapeutic targets and prognostic biomarkers in KIRC.

## Introduction

Kidney cancer, also known as renal cell carcinoma, is a serious and potentially life-threatening condition that affects millions of people worldwide [1–3]. The most common type of kidney cancer is clear cell renal carcinoma (KIRC), which accounts for approximately 70% of all cases [4]. KIRC arises from the lining of the small tubes in the kidney that filter blood and remove waste [5]. It typically begins as a small, bean-shaped mass within the kidney and can grow to invade surrounding structures or spread to other parts of the body [6]. Several factors can increase the risk of developing KIRC, including age, tobacco use, obesity, high blood pressure, and a family history of kidney cancer [7]. Symptoms of KIRC may include blood in the urine, persistent pain in the back or side, and unexplained weight loss [8]. However, many people with KIRC may not experience any symptoms until the cancer has advanced. In 2022, KIRC remained a global health concern, with varying states of prevalence and management strategies around the world. High-income countries continued to have better access to advanced diagnostics and therapies, while low-income regions faced challenges in early detection and treatment [9].

The diagnosis of KIRC typically involves a combination of imaging tests, such as ultrasound, CT scan, MRI or PET scan, and a biopsy to confirm the presence of cancerous cells [10]. Once a diagnosis of KIRC is confirmed, the stage of the cancer can be determined, which will guide treatment decisions [11]. Early-stage KIRC can often be treated with surgery to remove the affected kidney, while more advanced cases may require additional treatments, such as radiation therapy, chemotherapy, or targeted therapy [12]. The prognosis for KIRC is largely dependent on the stage of the cancer at the time of diagnosis [13]. However, advanced stages KIRC patients have a lower survival rate. The outlook for KIRC has improved in recent years with the development of new treatments, such as immunotherapy, which harnesses the body’s immune system to target cancer cells [11].

Despite recent advances in the diagnosis and treatment of KIRC, many patients continue to experience poor outcomes. One of the reasons for this is that the current biomarkers used to diagnose and monitor KIRC are limited in their ability to predict disease progression, treatment response, and overall patient survival. Therefore, there is an urgent need to find new molecular biomarkers via a combined computational and experimental approach [14] that can provide more accurate information about KIRC and help guide personalized treatment decisions. Currently, the most widely used molecular biomarker for KIRC is the von Hippel-Lindau (VHL) gene mutation, which is present in up to 70% of sporadic KIRC cases [15,16]. VHL mutation status is often used to stratify patients into different risk groups and guide treatment decisions. However, not all KIRC patients have VHL mutations, and other genetic mutations and alterations may play a role in disease progression and treatment response. Therefore, the exploration of new biomarkers for KIRC, including analyzing gene expression profiles, DNA methylation patterns, and protein biomarkers is urgently required.

Matrix metalloproteinases (MMPs) are enzymes responsible for breaking down several protein components of the extracellular matrix [17]. In cancer cells, the MMP family causes damage to the extracellular matrix and basement membrane, playing a vital role in tumor invasion and metastasis by activating growth factors and enhancing angiogenesis [18]. Moreover, MMPs influence immune responses in cancer cells by creating an immunosuppressive microenvironment [19]. This immunosuppressive microenvironment is a critical factor in cancer progression. Within tumor tissues, immune cells often become exhausted or are manipulated by cancer cells to promote an immunosuppressive state [20]. Regulatory T cells and myeloid-derived suppressor cells further inhibit immune responses. This microenvironment allows cancer cells to evade detection and destruction by the immune system, facilitating tumor growth, metastasis, and treatment resistance [20]. The MMP family is associated with tumor proliferation, differentiation, and angiogenesis, making them suitable therapeutic targets and molecular biomarkers for different cancers, such as gastric and head and neck cancers [21–24]. Previous research has also explored the involvement of the MMP gene family in renal carcinoma. For instance, studies have shown that MMP2 and MMP9 are up-regulated in clear cell renal cell carcinoma (ccRCC) samples [25]. Furthermore, one investigation employing immunohistochemistry (IHC) revealed a significant association between overexpressed MMP2 and MMP9 expression and distinct histological subtypes within ccRCC [26]. Additionally, elevated MMP2 and MMP9 expression levels were linked to unfavorable prognostic factors, including reduced patient survival in ccRCC [26]. Another study, employing radioactive-labeled riboprobe in situ hybridization and immunohistochemistry, provided evidence of MMP2 and MMP9 overexpression in RCC at both the mRNA and protein levels [27]. However, the role of other MMP family members is not yet explored comprehensively in KIRC patients.

The objective of this article is to investigate the expression, methylation, prognosis, mutation, and protein interaction, as well as the functional enrichment, related signaling pathways, therapeutic drugs, and perform experimental validation of the MMP family in KIRC. By examining these factors, we hope to accurately determine that a few hub genes from the MMP family can be utilized as both therapeutic targets and diagnostic biomarkers for KIRC.

## Materials and Methods

### KIRC and normal control tissue sample collection

Following the approval of the ethics committee at the Gomal University, Dera Ismail Khan, Pakistan, we conducted a prospective collection of 17 pairs of KIRC tissues and corresponding normal tissues from patients who visited the Institute of Nuclear Medicine, Oncology and Radiotherapy Hospital and Ayub Medical Complex between August 2022 and May 2023. Prior to their participation, all individuals provided informed consent by signing consent forms. All patients included in the study were diagnosed with KIRC cancer and had not undergone adjuvant or neoadjuvant therapy prior to surgery.

### List of the analyzed MMP genes in KIRC

Following 24 genes of MMP family, including MMP1, MMP2, MMP3, ILF3, MMP7, MMP8, MMP9, MMP10, MMP11, MMP12, MMP13, MMP14, MMP15, MMP16, MMP17, MMP19, MMP20, MMP21, MMP23B, MMP24, MMP25, MMP26, MMP27, MMP28 were analyzed in the present study for identifying and validating hub genes (molecular biomarkers) in KIRC patients.

### PPI of the MMP family members and hub gene identification

The STRING database is widely recognized as one of the most comprehensive and up-to-date databases for protein-protein interactions (PPIs) available today [28]. It provides a highly integrated platform for researchers to explore the complex interactions and functions of proteins in a vast array of biological systems, including humans, yeast, and bacteria. Advanced visualization tools and interactive networks also help to provide an intuitive representation of protein interactions and functions [28]. In this work, we used STRING web resource for constructing the PPI network of the MMP proteins family with default setting.

Cytoscape software [29] is a powerful tool used by researchers to analyze protein-protein interaction networks. This software allows users to visualize the complex networks of interactions between proteins and identify key players within the network. The Cytohubba plugin application [30] of the Cytoscape platform was used to identify hub genes from the constructed PPI based on the degree method.

### mRNA and protein expression profiling of hub genes in TCGA datasets

UALCAN is a user-friendly and publicly accessible database that provides gene expression analysis of cancer data from The Cancer Genome Atlas (TCGA) [31]. It allows researchers to easily explore gene expression levels, patient survival, and other clinical and molecular features across various cancer types. The database provides information such as gene expression quantification, correlations between gene expression and clinical data, pathway analysis, and gene ontology analysis. UALCAN was used in this work for mRNA and protein expression profiling of the hub genes across KIRC samples relative to controls.

### mRNA expression validation and survival analysis of hub genes using additional TCGA detests

GEPIA [32], OncoDB [33], and GENT2 [34] are web-based platforms for analyzing gene expression patterns in cancer. These databases use data from public datasets such as TCGA and GTEx to provide users with a comprehensive platform for gene expression analysis. These platforms offer a range of analysis tools including differential gene expression analysis, survival analysis, correlation analysis, and pathway analysis. The user-friendly interface of these three databases allows researchers to easily customize and edit data visualizations such as scatter plots, heatmaps, and bar graphs. In this work, GEPIA, OncoDB, and GENT2 databases were used for the expression validation analysis of the hub genes across KIRC samples relative to controls. Moreover, the GEPIA database was further utilized for survival analysis as well.

### Methylation analysis of hub genes using TCGA datasets

MEXPRESS [35] and OncoDB [33] databases were used in the present work for the methylation analysis of the hub genes across KIRC samples relative to controls.

### Development of hub genes-based prognostic model

The least absolute shrinkage and selection operator (Lasso) and multivariate Cox proportional hazard regression analysis were further developed to construct the prediction model with the “survival” package in R language [36]. In this analysis, the TCGA-KIRC dataset as the training dataset, while the GSE22541, GSE167573, and E_MTAB_1980 datasets served as the validation datasets. In this analysis, positive coefficients indicate increased risk of an event (e.g., death), while negative coefficients suggest reduced risk. Their magnitude signifies the impact of variables on hazard rates, aiding in building prognostic models for survival outcomes. The formula of the prognostic model of KIRC patients’ prognosis was as follows: risk score = the sum of the multivariate Cox regression coefficient variation of each mRNA.

### Mutational analysis of hub genes using the TCGA dataset

cBioPortal is an open-access database that houses cancer genomics data from cancer patients [37]. The database provides a user-friendly interface to search genomic profiles of cancers from many different sources. Researchers can use cBioPortal for different cancers and molecular data types to explore patients’ and cell lines’ genomic profiles. The main goal of cBioPortal is to provide an easy-to-use platform that enables researchers to study genomic alterations in different cancers and identify the molecular changes underlying tumor progression and drug resistance. In this work, this tool was used for the mutational analysis of the hub genes in TCGA KIRC samples.

### Functional enrichment analysis

To annotate the gene(s) of interest functionally, researchers relied on the Gene Ontology (GO) analysis. On the other hand, the KEGG (Kyoto Encyclopedia of Genes and Genomes) approach was considered to interpret the biological pathways of the user-defined genes [38]. The GO and KEGG analysis of the hub genes was performed using the GSEA program [39].

### Immune cell infiltration analysis

The TIMER2 (Tumor Immune Estimation Resource 2.0) database is a valuable resource for researchers working in the field of cancer immunology, as it offers a comprehensive analysis of the immune infiltrates in various types of tumors [40]. It provides extensive data on immune infiltration in various cancers, along with gene expression, somatic mutation, and clinical data. TIMER2 utilizes the CIBERSORT algorithm, which allows users to accurately estimate the immune cell populations in the tumor microenvironment [40]. In this research, levels of immune cell infiltration in KIRC were plotted against hub gene expression.

### Hub genes’ drug prediction analysis

Drugbank is a comprehensive database containing detailed information about drugs, their targets, and their interactions [41]. It is a widely used resource for researchers, clinicians, and drug developers around the world. Drugbank contains information on over 11,000 drugs, including their chemical structures, mechanisms of action, side effects, indications, and clinical studies. We used the DrugBank database to uncover a variety of drugs associated with the identified hub genes because we believe that the identified hub genes could be promising therapeutic targets.

### Cell lines

Cell lines: Human KIRC cell lines (786-O and A-498), and normal renal tubular epithelial cell line (HK-2) were purchased from the American Type Culture Collection (ATCC, USA) and cultivated in accordance with the manufacturer’s instructions.

### DNA and RNA extraction

Total RNA extraction from both clinical KIRC samples, cell lines and normal control samples, and cell lines was done by isopycnic centrifugation as described previously [42]. The DNA extraction was done following the organic method [43]. The quality of the extracted RNA and DNA was checked by a 2100 Bioanalyzer (Agilent Technologies, Germany).

### Targeted bisulfite sequencing (targeted bisulfite-seq) analysis

DNA samples were sent to Beijing Genomics Institute (BGI) Company for RNA-seq bisulfite-seq analysis. Following targeted bisulfite-seq analysis, methylation values were normalized as beta values. The obtained beta values against hub genes in KIRC samples, cell lines and normal control samples, and cell lines were compared to identify differences in the expression and methylation levels.

### RT-qPCR validation analysis

The specific protocols are as follows: First, the PrimeScript™ RT reagent kit (Takara, Japan) was used for reverse transcription of the extracted RNA from clinical KIRC samples, cell lines, and normal control samples and cell lines into complementary DNA. Then, the RT-qPCR was carried out on an ABI ViiA 7 Real Time PCR System (Thermo Fisher, USA) with a SuperReal SYBR Green Premix Plus (Tiangen Biotech, China) as a fluorescent dye. GAPDH was chosen as the internal reference in the present study. All the experiments were in triplicate independently. All the primers of each hub gene are shown as follows. The 2^−ΔΔCt^ method was employed to evaluate the relative expression of each hub gene [44].

GAPDH-F 5-ACCCACTCCTCCACCTTTGAC-3, GAPDH-R 3-CTGTTGCTGTAGCCAAATTCG-5 [45].

MMP2-F 5′-CTCAGATCCATGGTGAGATCT, MMP2-R 5′-CTTTGGTTCTCCAGCTTCAGG-3′ [46].

MMP9-F 5′-GAGTGGCAGGGGGAAGATGC-3′, MPP9-R 5′-CCTCAGGGCACTGCAGGATG-3′ [47].

MMP12-F 5′-TTTCTTCCATATGGCCAAGC-3′, MPP12-R 5′-GGTCAAAGACAGCTGCATCA-3′ [48].

MMP16-F 5′-TCTGTCTCCCTTGAAATA-3′, MMP16-R 5′-ACCCTCATGACTTGATAACC-3′ [49].

### Conventional PCR and sanger sequencing for mutation detection

In this study, conventional PCR was employed to target Exon 13 of MMP2 and MMP9 genes for the detection of genetic mutations, if present. The primer pairs used for both genes were sourced from previous studies cited in the medical literature [50,51]. A DNA amount of 100 ng obtained from 17 KIRC (Kidney Renal Clear Cell Carcinoma) patients was initially subjected to PCR amplification using the optimized cyclic conditions provided in those referenced studies. The PCR master mix used was Thermo Scientific 2X (lot No. 00097068). Following amplification, the PCR products were sent to Macrogen Company for bidirectional Sanger sequencing analysis.

### Statistics details

For enrichment analysis, we used the Fisher’s Exact test for computing statistical difference [52]. Correlational analyses were carried out using the Pearson method. For comparisons, a student *t*-test was adopted in the current study. All the analyses were carried out in R version 3.6.3 software.

## Results

### PPI network of the MMP family members and hub genes recognition

**Figure 1:**
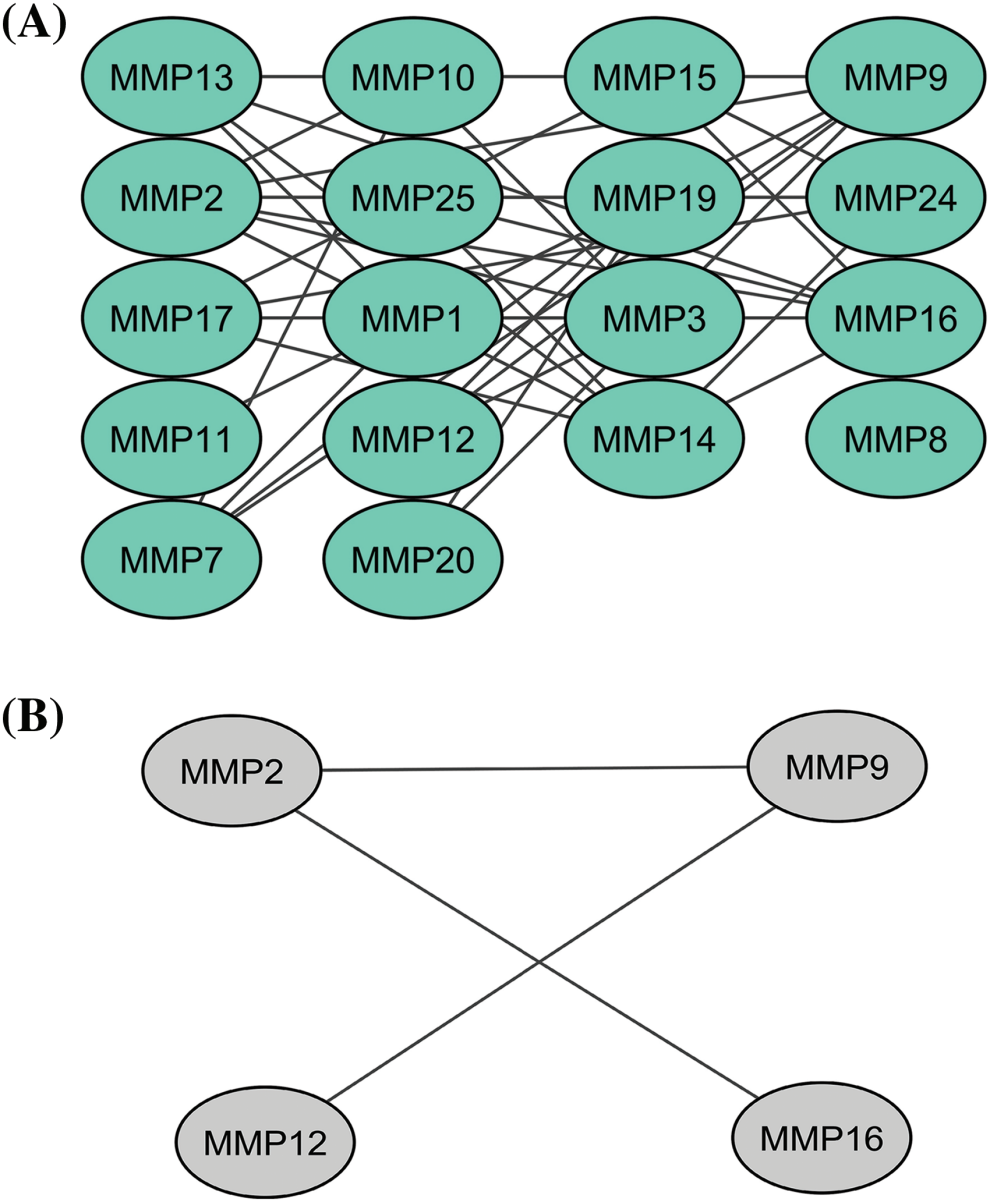
A PPI network of the MMP family proteins and a PPI network of the identified hub genes. (A) A PPI network of the MMP family proteins, and (B) A PPI network of identified four hub genes.

In total 24 proteins belonging to the MMP family were evaluated for constructing the PPI network through the STRING database. As highlighted in Fig. 1A, the constructed PPI consisted of 24 nodes, where nodes and edges represent proteins and protein–protein association, respectively. Further, the constructed PPI was analyzed using the Cytohubba plugin application of the Cytoscape software to identify hub genes based on the degree method. This analysis revealed the MMP2 (matrix metallopeptidase 2), MMP9 (matrix metallopeptidase 9), MMP12 (matrix metallopeptidase 12), and MMP16 (matrix metallopeptidase 16) genes were the hub genes having highest degree scores (Fig. 1B).

### Expression and correlation analysis of MMP2, MMP9, MMP12, and MMP16 with different clinical variables among KIRC patients

**Figure 2:**
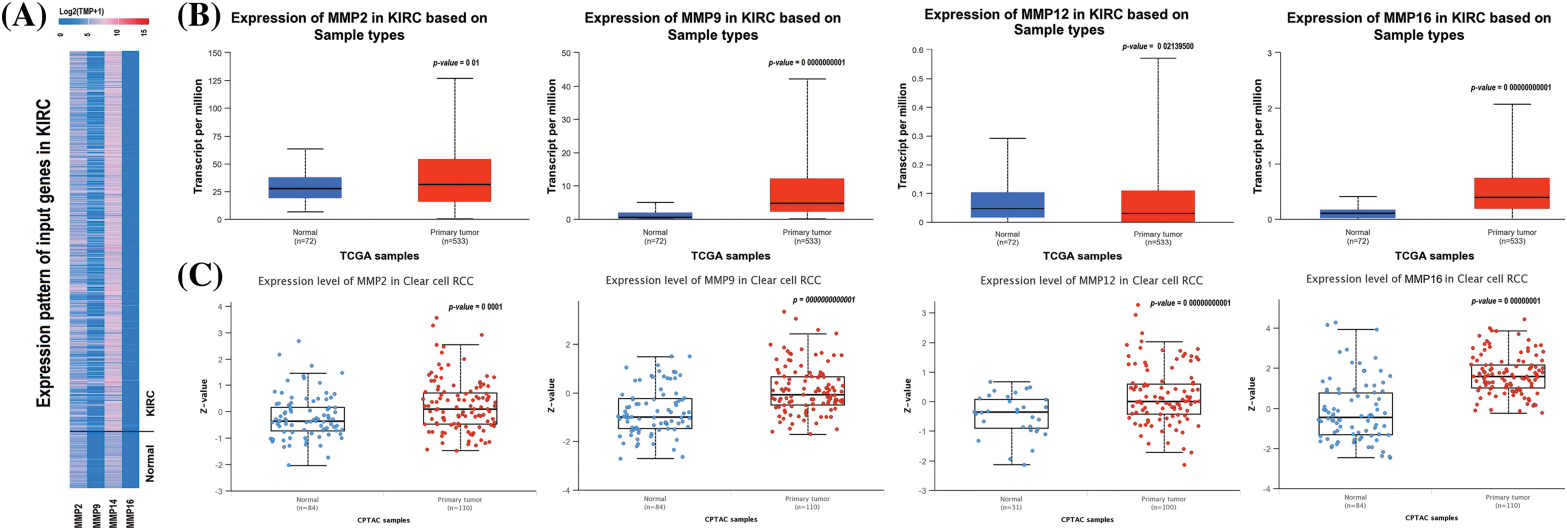
mRNA and protein expression profiling of the MMP2, MMP9, MMP12, and MMP16 via UALCAN. (A) A heatmap of MMP2, MMP9, MMP12, and MMP16 hub genes expression in KIRC sample group as compare to normal control group, (B) Box plot presentation of MMP2, MMP9, MMP12, and MMP16 hub genes mRNA expression in KIRC sample group as compare to normal control group, and (C) Box plot presentation of MMP2, MMP9, MMP12, and MMP16 hub genes protein expression in KIRC sample group as compare to normal control group.

In order to analyze the expression levels of MMP2, MMP9, MMP12, and MMP16, we utilized the KIRC TCGA dataset via UALCAN tool. Results of this analysis revealed a notable increase in expression at both mRNA and protein levels for all four MMP hub genes (MMP2, MMP9, MMP12, and MMP16) in KIRC samples compared to controls (as seen in Figs. 2A–2C). All these differences were found to be statistically significant with a *p*-value less than 0.05.

**Figure 3:**
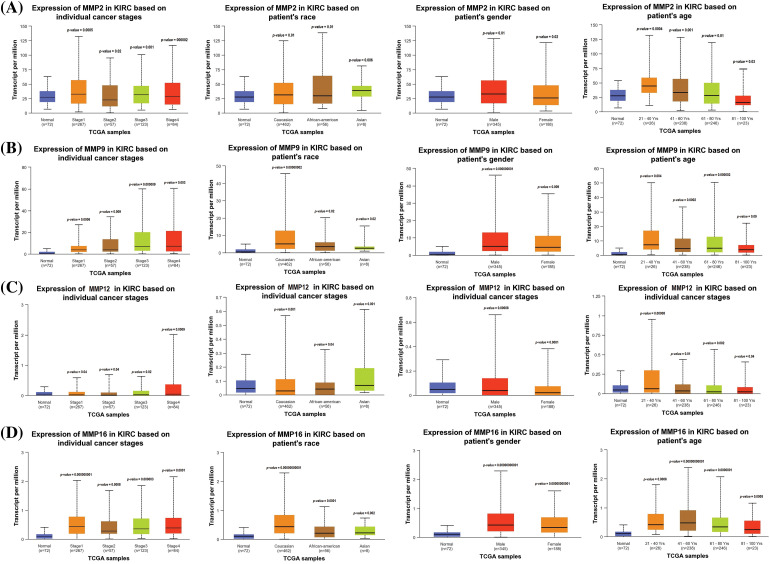
Expression profiling of MMP2, MMP9, MMP12, and MMP16 in KIRC samples of different clinical variables as compare to controls via UALCAN. (A) Expression profiling of MMP2 in KIRC samples of different clinical variables as compare to normal controls, (B) Expression profiling of MMP9 in KIRC samples of different clinical variables as compare to normal controls, (C) Expression profiling of MMP12 in KIRC samples of different clinical variables as compare to normal controls, and (D) Expression profiling of MMP16 in KIRC samples of different clinical variables as compare to normal controls.

Additionally, we delved further into the potential implications of the dysregulation of these genes in KIRC patients with various clinicopathological parameters. Through the use of UALCAN, relevant expression profiles of the hub genes were analyzed in KIRC patients with different clinical variables, such as cancer stage, race, gender, and age and normal controls. Results of this analysis showed a significant increase in MMP2, MMP9, MMP12, and MMP16 levels in KIRC patients across different cancer stages, races, genders, and age groups when compared to normal controls (Fig. 3).

### Expression verification and survival analyses of MMP2, MMP9, MMP12, and MMP16

**Figure 4:**
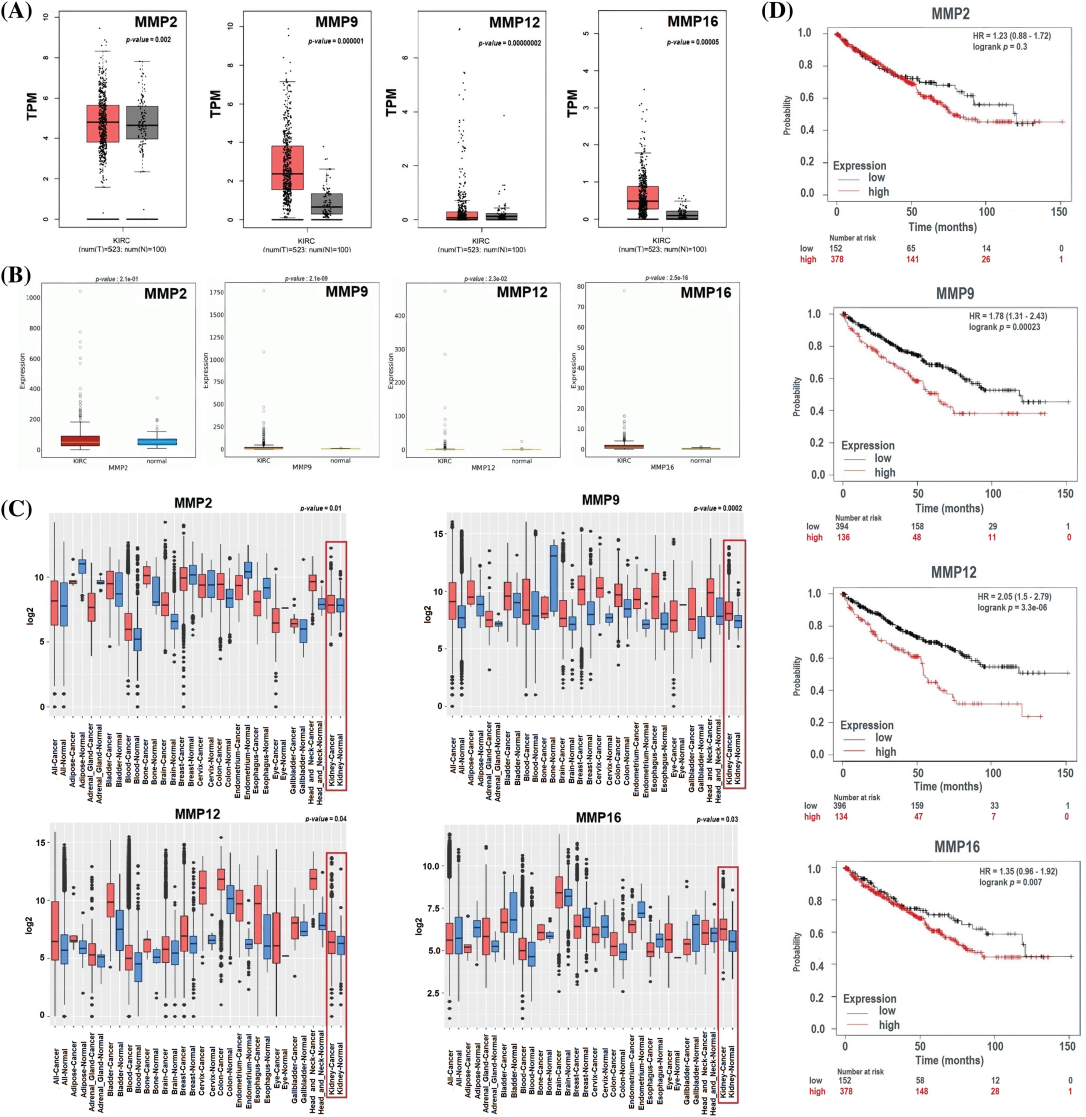
Expression validation and survival analysis of MMP2, MMP9, MMP12, and MMP16. (A) Expression validation of MMP2, MMP9, MMP12, and MMP16 in KIRC samples as compare to normal controls via GEPIA database, (B) Expression validation of MMP2, MMP9, MMP12, and MMP16 in KIRC samples as compare to normal controls via OncoDB database, (C) Expression validation of MMP2, MMP9, MMP12, and MMP16 in KIRC samples as compare to normal controls via GENT2 database, and (D) Survival analysis of MMP2, MMP9, MMP12, and MMP16 in KIRC and normal samples via GEPIA database.

To ensure the credibility of their findings, we used three additional databases, namely GEPIA, OncoDB, and GENT2, for expression verification analysis. Specifically, we assessed the expression levels of the MMP2, MMP9, MMP12, and MMP16 and their impact on the survival of KIRC patients (Fig. 4). Results of the analysis revealed significantly (*p*-value < 0.05) higher mRNA expression levels of MMP2, MMP9, MMP12, and MMP16 in KIRC samples compared to healthy ones (Figs. 4A–4C). Additionally, the GEPIA database was utilized to investigate the hub genes’ effect on the OS of the KIRC patients. Results of the analysis revealed a significant (*p* < 0.05) correlation between dysregulated MMP9, MMP12, and MMP16, and poor OS, while insignificant (*p* > 0.05) in case of MMP2 across KIRC patients (Fig. 4D).

### Development of hub genes-based prognostic model

**Figure 5:**
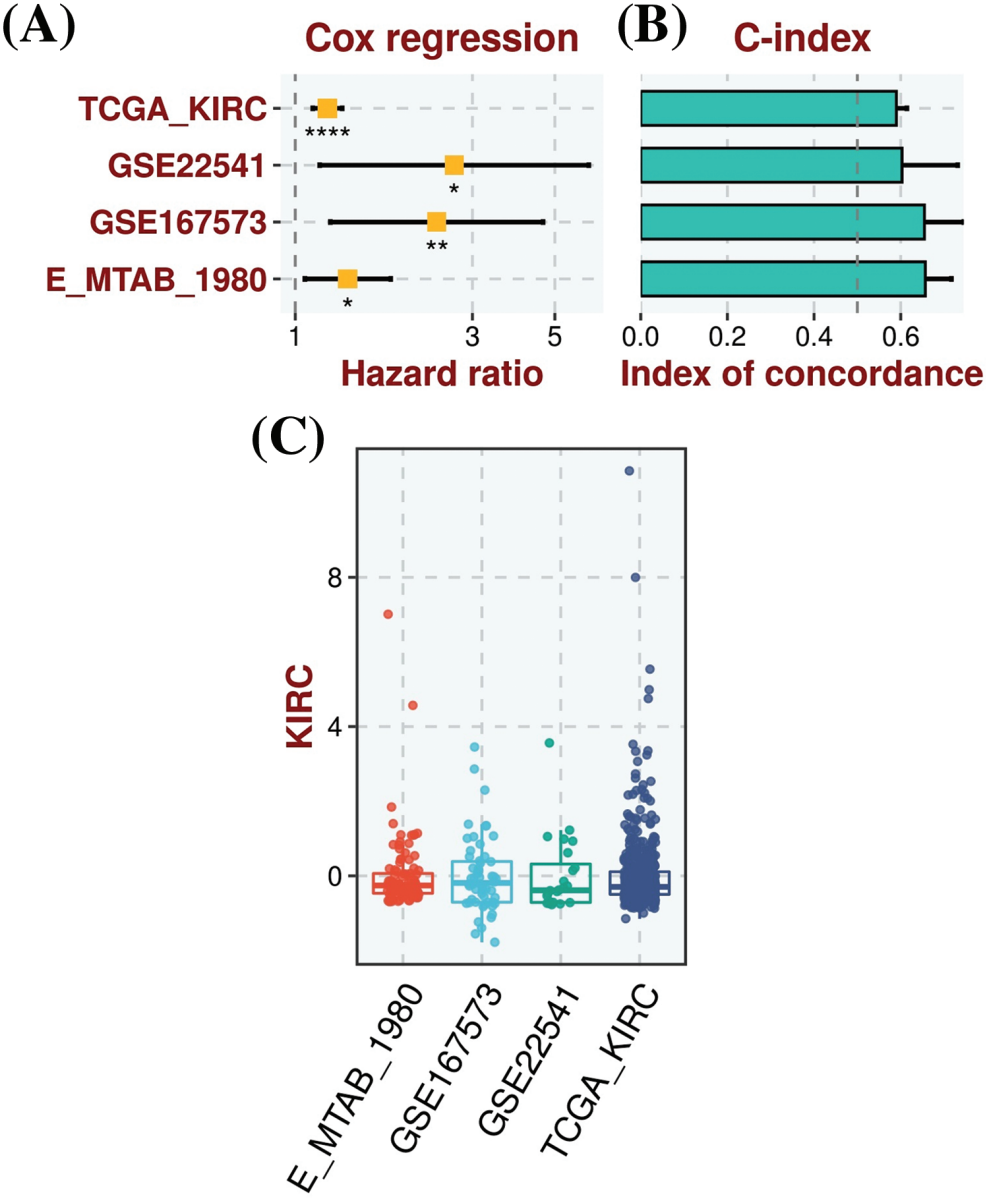
Construction of the hub genes (MMP2, MMP9, MMP12, and MMP16) based prognostic model. (A) Univariate Cox regression analysis depicting the association of gene expression with patient survival in KIRC, (B) C-index scores, illustrating the model’s discriminatory ability to predict patient outcomes, with higher values indicating better performance, and (C) Risk scores, representing individual patient risk scores based on hub gene expression levels, where higher scores correlate with poorer prognosis. *Sign is used to denote the significance level of hazard ratio (HR), *p < 0.05; **p < 0.01; ****p < 0.0001.

For the analysis of the prognostic model based on MMP2, MMP9, MMP12, and MMP16 genes, we employed the TCGA-KIRC dataset as the training dataset, while the GSE22541, GSE167573, and E_MTAB_1980 datasets served as the validation datasets. To construct a prognostic model, we used a stepwise Cox regression model that incorporated the parameters of hazard ratio, c-index, and risk score. By evaluating our predictive prognostic model using the c-index, we determined that this model significantly, effectively, and robustly assessed the prognosis of KIRC patients in all the analyzed datasets (Fig. 5).

### DNA mutations and promoter methylation on the dysregulation of the hub genes

**Figure 6:**
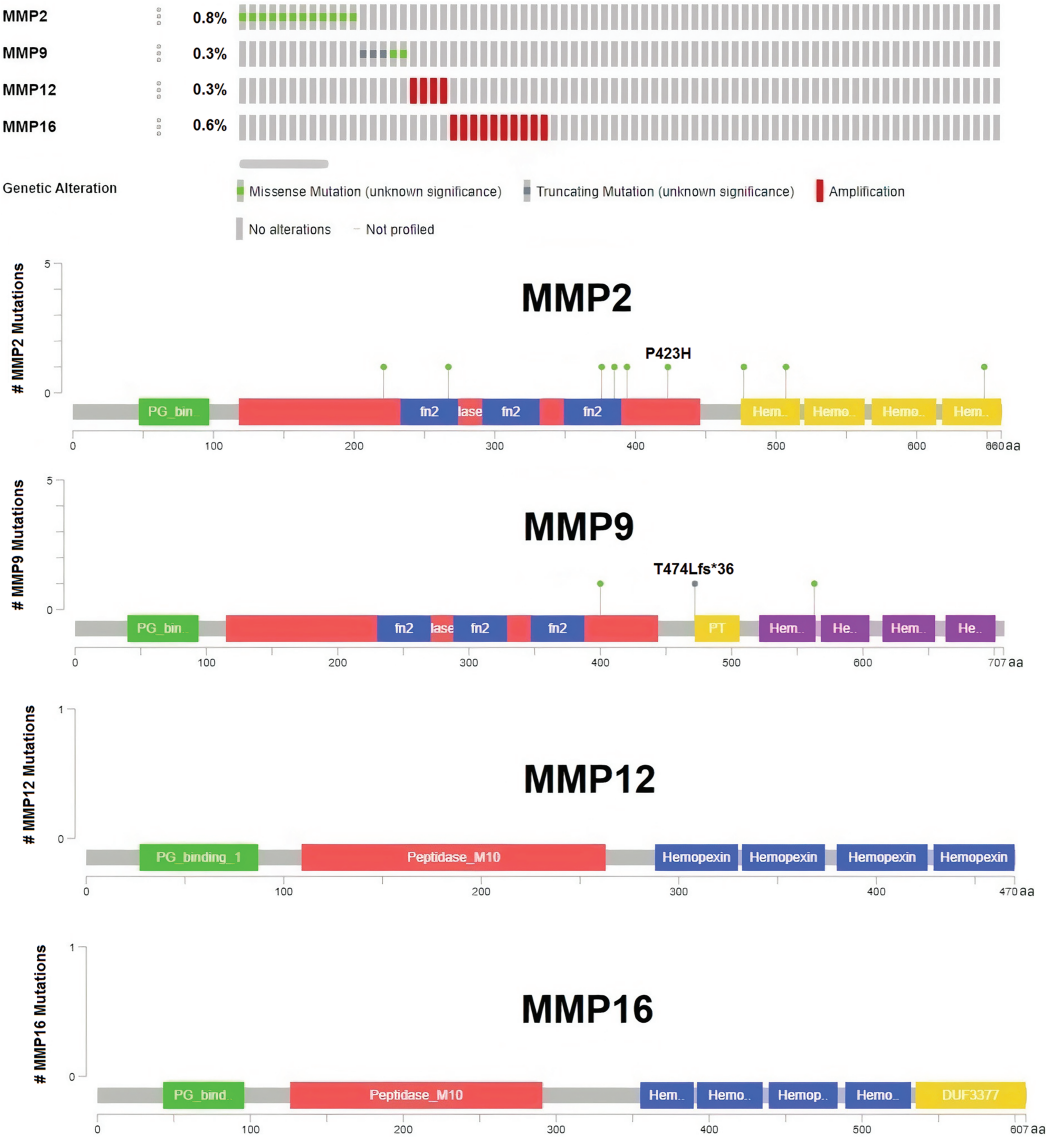
DNA mutation analysis of MMP2, MMP9, MMP12, and MMP16 in The Cancer Genome Atlas (TCGA) KIRC samples via the cBioPortal databases.

Significant correlations were observed between the dysregulation of MMP2, MMP9, MMP12, and MMP16 expressions and various clinical parameters of KIRC, as well as worse overall survival. Therefore, investigating the potential regulatory mechanisms of these hub genes’ overexpression could hold clinical significance. Initially, the cBioPortal database was utilized to identify genetic mutations in the TCGA KIRC cohorts. Low occurrence frequencies of genetic alterations were noted in MMP2 (0.8%), MMP9 (0.3%), MMP12 (0.3%), and MMP16 (0.7%), indicating their limited involvement in hub gene expression regulation (Fig. 6).

**Figure 7:**
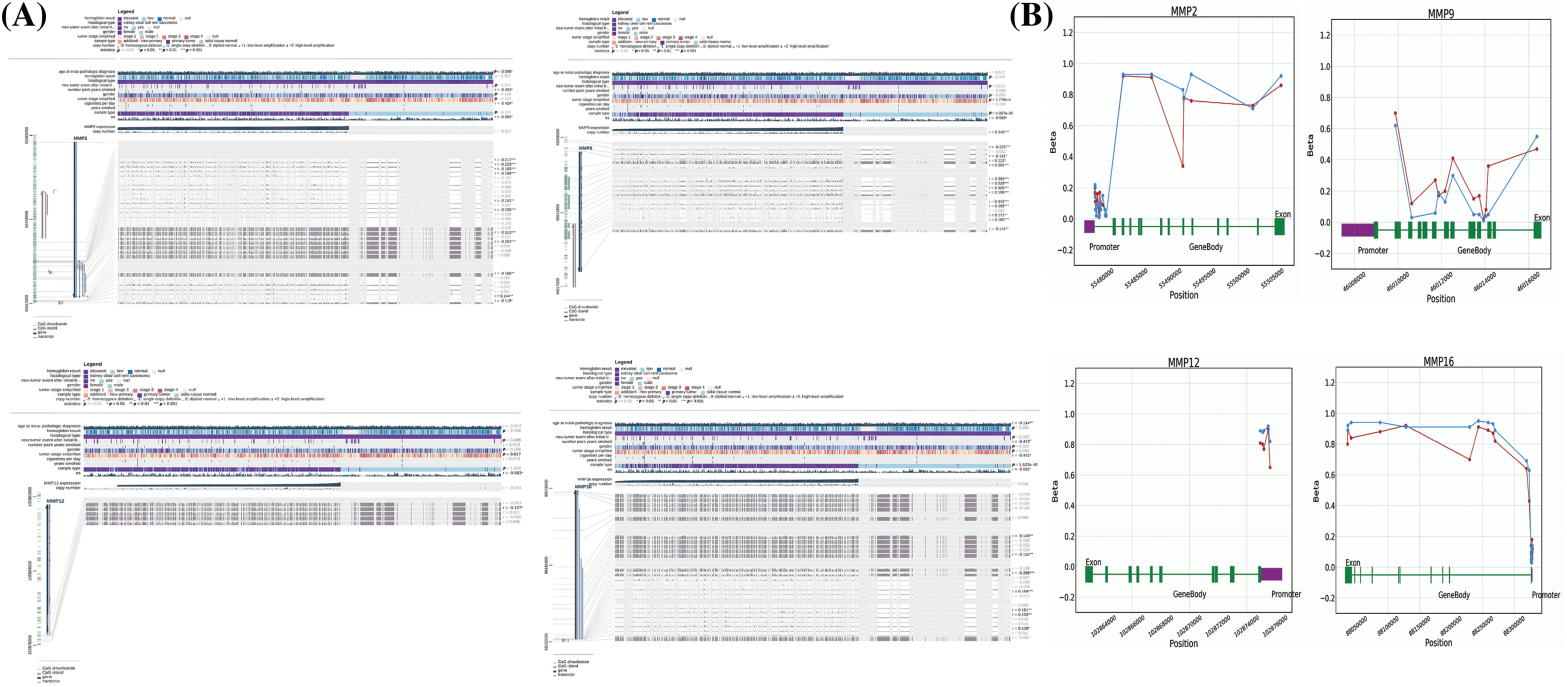
Methylation status exploration of MMP2, MMP9, MMP12, and MMP16 in KIRC samples as compare to normal controls via MEXPRESS and OncoDB in KIRC and normal samples. (A) Methylation status exploration of MMP2, MMP9, MMP12, and MMP16 in KIRC samples as compare to normal controls via MEXPRESS, and (B) Methylation status exploration of MMP2, MMP9, MMP12, and MMP16 in KIRC samples as compare to normal controls via OncoDB.

Next, the examination of hub gene promoter methylation levels was conducted using MEXPRESS and OncoDB. The results showed that MMP2, MMP9, MMP12, and MMP16 promoter regions in KIRC samples were hypomethylated compared to non-cancer samples (as shown in Figs. 7A and 7B).

### GO and biological pathways analysis

**Figure 8:**
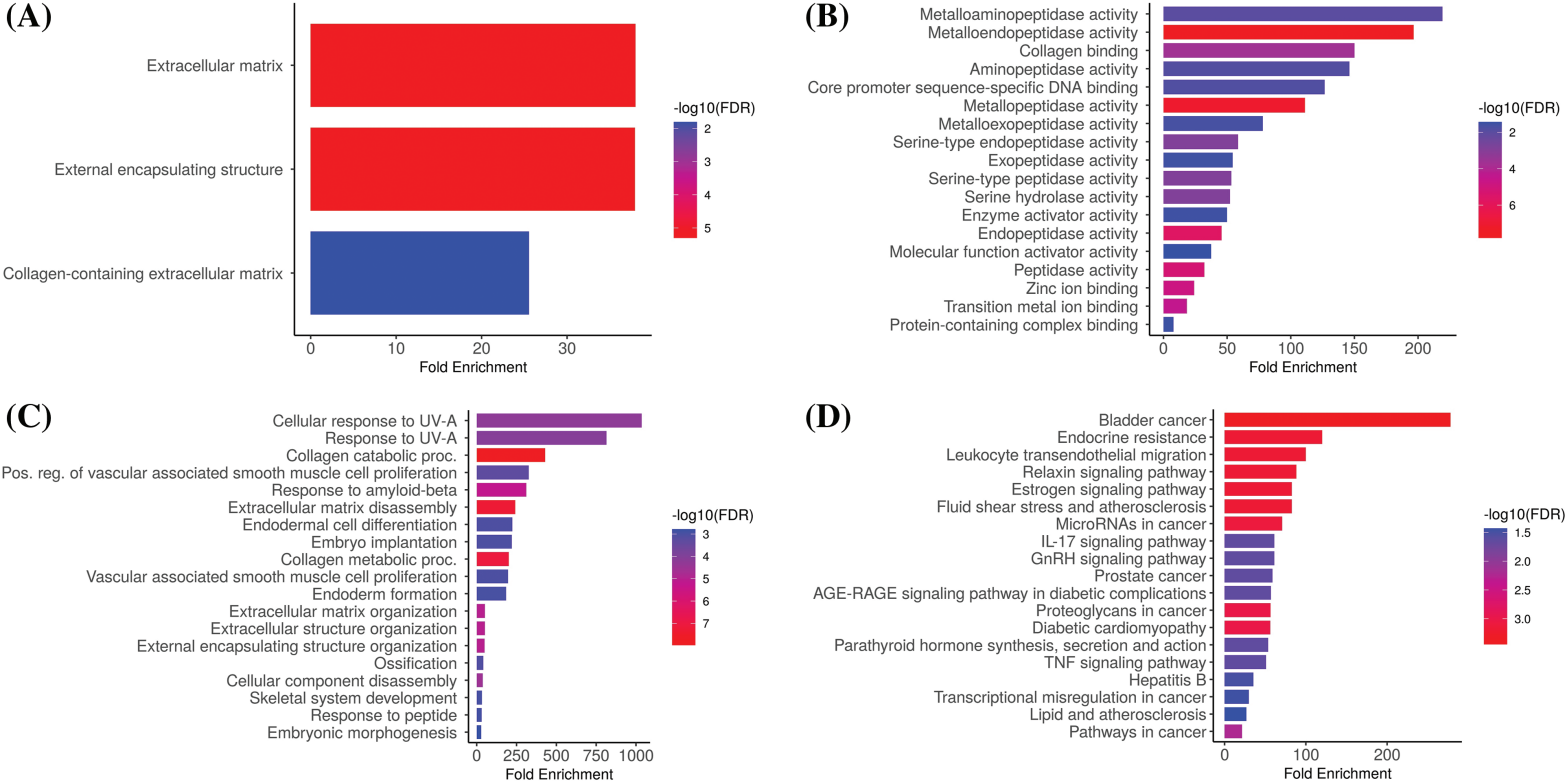
Gene enrichment analysis of MMP2, MMP9, MMP12, and MMP16. (A) MMP2, MMP9, MMP12, and MMP16 associated CC terms, (B) MMP2, MMP9, MMP12, and MMP16 BP terms, (C) MMP2, MMP9, MMP12, and MMP16 associated MF terms, and (D) MMP2, MMP9, MMP12, and MMP16 associated KEGG terms.

We analyzed hub genes to figure out their GO and KEGG pathways in KIRC. In the CC, “Extracellular matrix, external encapsulating structure, and collagen-containing extracellular matrix”, etc., terms were significantly associated with the MMP2, MMP9, MMP12, and MMP16 (Fig. 8A). Concerning MF, the “Metalloaminopeptidase activity, Metallendopeptidase activity, and collagen binding”, etc., terms were closely associated with the MMP2, MMP9, MMP12, and MMP16 (Fig. 8B). In BP, some vital functions including “Cellualr response to UV-A, response to UV-A, and collagen catabolic proc”, etc., terms were significantly associated with the MMP2, MMP9, MMP12, and MMP16 (Fig. 8C). Moreover, MMP2, MMP9, MMP12, and MMP16-enriched KEGG pathways include “Bladder cancer, endocrine resistance, and relaxin signaling pathway”, etc., (Fig. 8D).

### Immune cells analysis of MMP2, MMP9, MMP12, and MMP16

**Figure 9:**
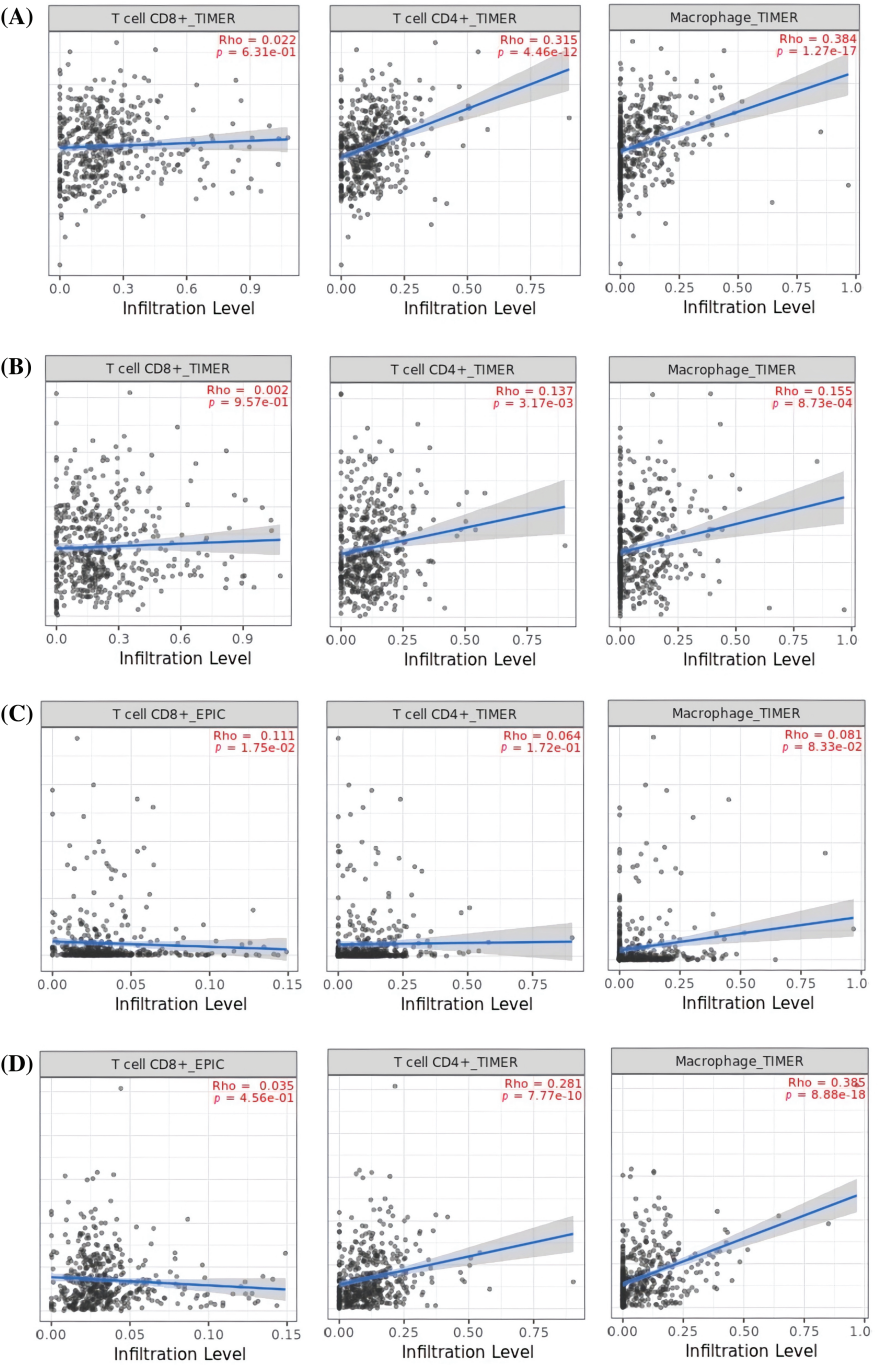
Correlation analysis of MMP2, MMP9, MMP12, and MMP16 hub genes expression with different immune cells (CD8+ T, CD4+ T, and Macrophages) infiltration level across KIRC samples. (A) MMP2, (B) MMP9, (C) MMP12, and (D) MMP16.

In order to reveal the links between the expression of MMP2, MMP9, MMP12, and MMP16 genes and the influx of immune cells (namely, CD8+ T, CD4+ T, and macrophages) in KIRC samples, TIMER was employed. The results showed a significant positive correlation (*p* < 0.05) between the infiltration of CD8+ T, CD4+ T, macrophages, and the expression levels of MMP2, MMP9, MMP12, and MMP16 genes in KIRC (Fig. 9).

### Drug prediction analysis of the MMP2, MMP9, MMP12, and MMP16

For KIRC patients suffering from cisplatin drug, the use of alternative drugs for medical treatment is the first option. Therefore, a selection of appropriate alternate candidate drugs is required. In the current study, via the DrugBank database, we explored some potential drugs that can reverse the gene expressions of MMP2, MMP9, MMP12, and MMP16 hub genes. Alvocidib, Estradiol, Capsaicin, Dronabinol, Acetaminophen, Estradiol, and Azacitidine drugs (Table 1) could be useful to target MMP2, MMP9, MMP12, and MMP16 hub genes once clinical significance is established via preclinical/clinical studies.

**Table 1:** DrugBank-based MMP2, MMP9, MMP12, and MMP16 associated drugs

Sr. No.	Hub gene	Drug name	Effect	Reference	Group
1	MMP2	Alvocidib	Decrease expression of MMP2 mRNA	A20630	Approved
		Estradiol		A21152	
2	MMP9	Capsaicin	Decrease expression of I MMP9 mRNA	A21513	Approved
		Dronabinol		A22085	
3	MMP12	Acetaminophen	Decrease expression of MMP12 mRNA	A20420	Approved
4	MMP16	Estradiol	Decrease expression of MMP16 mRNA	A21179	Approved

### Cell lines and clinical sample-based validation of MMP2, MMP9, MMP12, and MMP16 expression

**Figure 10:**
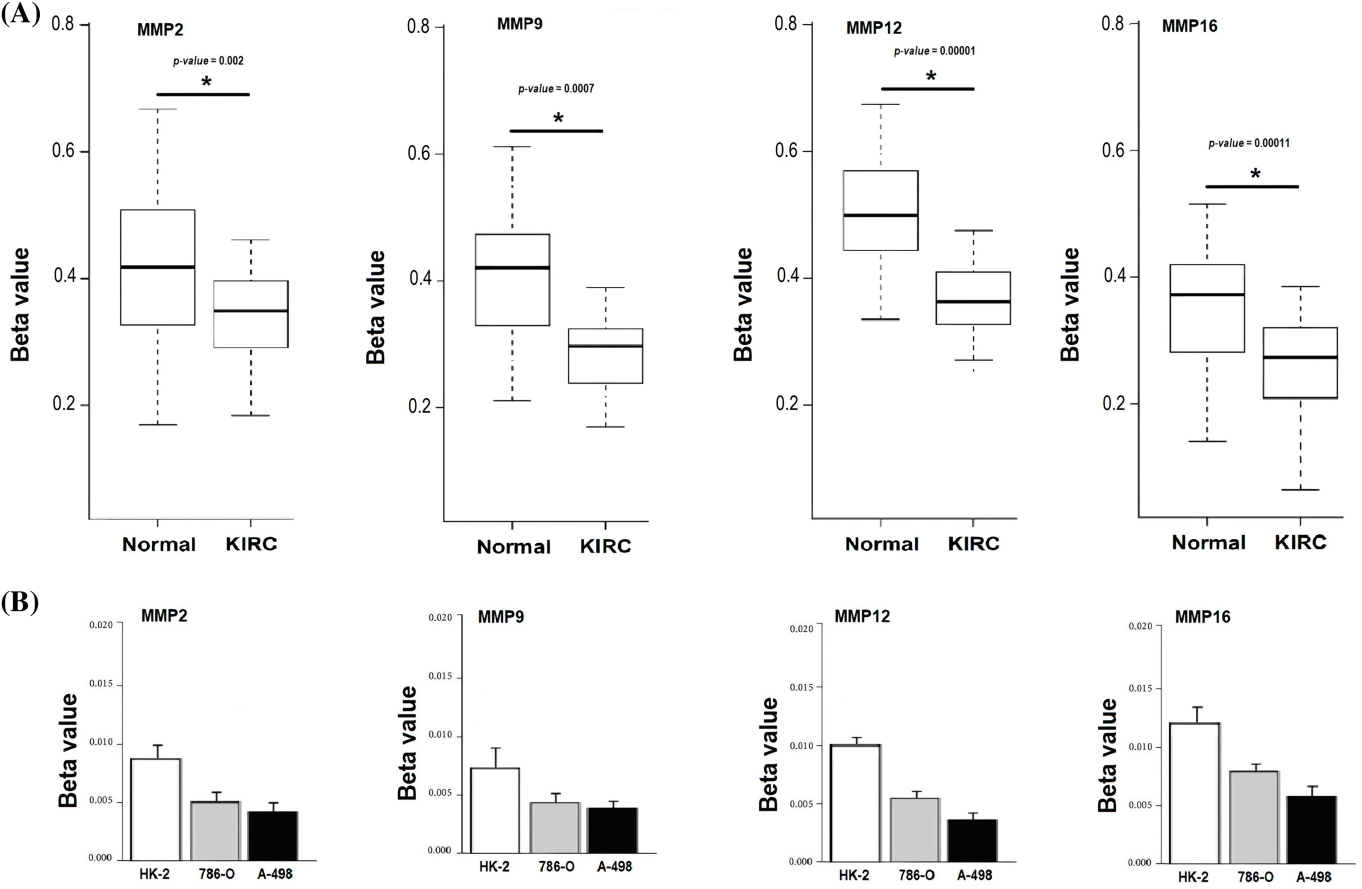
Validating MMP2, MMP9, MMP12, and MMP16 promoter methylation levels on clinical KIRC samples and cell lines (786-O and A-498) paired with controls via targeted bisulfite-seq analysis. (A) Beta values based promoter methylation based validation of MMP2, MMP9, MMP12, and MMP16 in clinical KIRC samples as compare to controls. *p < 0.05; (B) Beta values based promoter methylation based validation of MMP2, MMP9, MMP12, and MMP16 in KIRC cell lines as compare to control cell line.

In the current study, we conducted bisulfite-seq analysis on a dataset comprising 17 KIRC clinical samples matched with control samples and evaluated two KIRC cell lines (786-O and A-498) alongside a normal renal tubular epithelial cell line, HK-2, to experimentally validate the methylation levels of the MMP2, MMP9, MMP12, and MMP16 genes. We employed a widely accepted quantitative metric, beta values for quantifying methylation level. Beta value is a continuous variable that represents the proportion of DNA molecules that are methylated at a specific CpG site within a genomic region. The results of bisulfite-seq analysis depicted in Figs. 10A and 10B highlight the significantly (*p*-value < 0.05) lower beta values for MMP2, MMP9, MMP12, and MMP16 in KIRC clinical samples and cell lines (786-O and A-498) when compared to normal control counterparts (Figs. 10A and 10B). This demonstrates the hypomethylation pattern associated with these genes in KIRC.

### RT-qPCR validation analysis of MMP2, MMP9, MMP12, and MMP16

To provide robust experimental validation for our bioinformatics findings, we conducted a RT-qPCR experiment. The aim was to measure the mRNA expression levels of the identified hub genes (MMP2, MMP9, MMP12, and MMP16) in KIRC clinical samples and cell lines (786-O and A-498) in comparison to normal controls. Our analysis involved a total of 17 KIRC clinical samples. Additionally, we examined KIRC cell lines, 786-O and A-498, as these *in vitro* models complemented our clinical data, providing insights into potential therapeutic strategies.

**Figure 11:**
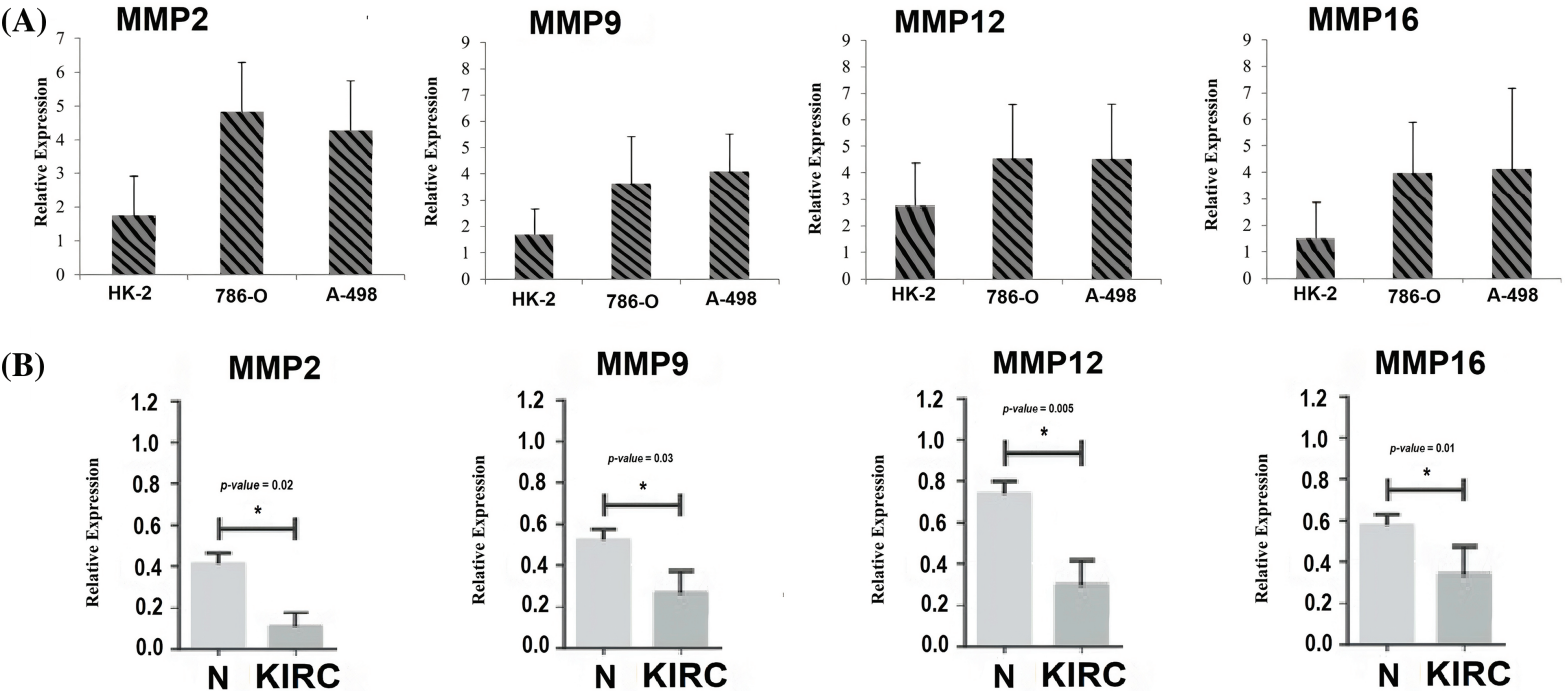
RT-qPCR validation of mRNA expression levels of MMP2, MMP9, MMP12, and MMP16 in clinical KIRC samples and cell lines (786-O and A-498) paired with controls. (A) RT-qPCR analysis of MMP2, MMP9, MMP12, and MMP16 in KIRC as compare to normal control cell line, and (B) RT-qPCR analysis of MMP2, MMP9, MMP12, and MMP16 in KIRC clinical samples as compare to normal control samples. The x-axis represents different groups, and the y-axis represents the relative expression of genes. *p < 0.05.

The results, as depicted in Fig. 11, demonstrated significant differences in the expression levels of all four hub genes within KIRC clinical samples and cell lines (786-O and A-498) as compared to their respective normal controls. Importantly, our findings confirmed the up-regulation of MMP2, MMP9, MMP12, and MMP16 in KIRC. This consistency with our predictions based on TCGA datasets analysis reinforces the reliability and relevance of our bioinformatics approach. The experimental validation of the overexpression of these hub genes strengthens their potential as diagnostic biomarkers and therapeutic targets in KIRC. These genes’ elevated expression levels in both clinical samples and cell lines indicate their consistent role in the molecular landscape of KIRC.

### Mutational analysis of MMP2 and MMP9 genes through conventional PCR and Sanger sequencing

After conducting a mutational analysis of hub genes utilizing TCGA datasets, a significant finding emerged. Among the samples from TCGA, only MMP2 and MMP9 genes exhibited mutations. As a result, further investigation was carried out to assess the mutation status of these genes in clinical samples obtained from KIRC patients. To accomplish this, conventional PCR and Sanger sequencing were employed to detect mutations in 17 clinical KIRC samples. However, the results yielded a surprising outcome, as no mutations were detected in either of these genes within the analyzed samples. This observation suggests that the mutational status of MMP2 and MMP9 genes may not play a significant role in the pathogenesis or progression of KIRC.

## Discussion

According to reports, the Zn-dependent protease, matrix metalloproteinase (MMP), has the ability to break down the extracellular matrix [53]. Several recent studies have highlighted the regulatory role of MMP in cancer-related activities such as tumor cell invasion, proliferation, metastasis, immunity, and angiogenesis [54,55]. Therefore, understanding the participation of MMP in the occurrence of cancer can be beneficial in developing targeted therapies. Various studies have investigated the role of the MMP family in several cancers like gastric and head and neck cancers [21–24]. However, there is no clear and systematic study of the MMP family as a therapeutic target and molecular biomarker in KIRC.

In the present study, firstly, a total of 24 MMP family genes were subjected to PPI construction. Then, the constructed PPI was screened out via the Cytohubba plugin of the Cytoscape software to identify hub genes. Based on the degree method, MMP2, MMP9, MMP12, and MMP16 genes were identified as the most prominent hub genes in KIRC. These hub genes exhibited a high degree of connectivity, signifying their central role in molecular interactions associated with KIRC. Their prominence suggests that MMP2, MMP9, MMP12, and MMP16 may play pivotal roles in the underlying mechanisms driving KIRC pathogenesis, making them attractive candidates for further downstream analyses. Furthermore, TCGA database and cell lines based experiments showed that MMP2, MMP9, MMP12, and MMP16 hub genes were significantly up-regulated across KIRC patients. Additionally, targeted bisulfite sequencing revealed a significant hypomethylation pattern within the promoters of these hub genes in KIRC, indicating potential epigenetic mechanisms driving their increased expression. Importantly, our findings also correlated the elevated expression of these hub genes with OS among KIRC patients, underlining their clinical significance in predicting disease outcomes. Therefore, the expression levels of MMP2, MMP9, MMP12, and MMP16 can serve as an independent risk factor for a poor prognosis of KIRC.

MMP2 is involved in tumor invasion, angiogenesis, and metastasis, which are key events in cancer progression [56]. MMP2 gene is being extensively studied as a biomarker gene because its activity can be detected in body fluids such as tissue, blood, urine, and saliva samples [57,58]. The detection of MMP2 in these samples can potentially serve as a diagnostic and prognostic tool in cancer. Additionally, the expression of MMP2 has been found to be associated with poor prognosis and reduced survival rates in cancer patients [59]. Therefore, targeting MMP2 expression and activity is a potential therapeutic approach to cancer treatment. In a nutshell, MMP2 gene is an important biomarker gene in cancer that can serve as a diagnostic, prognostic, and therapeutic target.

MMP9, also known as Matrix metalloproteinase-9, is a biomarker gene that has gained attention in the field of cancer research in recent years [60]. MMP9 gene is involved in processes such as cell proliferation, migration, and invasion, which are crucial in cancer progression [61]. It has been found to be up-regulated in various types of cancer, including breast, lung, colorectal, and head and neck cancers [62–64]. The overexpression of MMP9 has been associated with poor prognosis and increased cancer aggressiveness, making it a potential prognostic biomarker in cancer [65]. Additionally, MMP9 has been shown to play a role in the development of resistance to chemotherapy, which could have implications for treatment selection in cancer patients [66]. Given the significance of MMP9 gene in cancer, it is being thoroughly studied as a potential therapeutic target. Targeting the activity of MMP9 has been shown to have beneficial effects in reducing tumor growth and metastasis.

The MMP12 gene encodes for the matrix metalloproteinase 12, a proteolytic enzyme that can degrade the extracellular matrix and facilitate cancer cell invasion and metastasis [67]. The overexpression of MMP12 is associated with poor prognosis and increased risk of recurrence in many types of cancer, including lung, colon, breast, and ovarian cancers [68–70]. Therefore, the identification of MMP12 as a potential biomarker of cancer has significant clinical implications in cancer diagnosis, prognosis, and treatment [71]. MMP12 inhibition can suppress cancer cell migration, invasion, and metastasis, making MMP12 an attractive therapeutic target in cancer treatment [72]. Several preclinical studies have demonstrated the potential benefits of targeting MMP12 in cancer therapy, and ongoing clinical trials are exploring the efficacy and safety of this strategy. Therefore, the MMP12 gene represents a promising biomarker and therapeutic target for cancer management, with the potential to improve patient outcomes and survival rates.

MMP16 gene is a key biomarker of cancer that plays a role in the invasive behavior of cancer cells [73]. MMP16 gene encodes for the matrix metalloproteinase 16, a protease that degrades the extracellular matrix and promotes cancer cell invasion and metastasis [74]. MMP16 is up-regulated in several types of cancers, including breast, lung, prostate, and colon cancer [75–77]. The overexpression of MMP16 is associated with poor prognosis and increased risk of metastasis in cancer patients [78]. Therefore, MMP16 has been identified as a potential therapeutic target and biomarker of cancer. Inhibition of MMP16 can reduce cancer cell invasion, migration, and metastasis, providing a promising therapeutic strategy for cancer treatment.

According to TIMER analysis, there is a notable association between the expression of MMP2, MMP9, MMP12, and MMP16 and the presence of immune cells (CD8+ T cells, CD4+ T cells, and macrophages). CD8+ T cells are known for their cytotoxic activity against cancer cells, while CD4+ T cells aid in orchestrating immune responses. Macrophages, on the other hand, exhibit dual roles, either promoting tumor growth or contributing to its regression, depending on their activation state [79]. However, if these cells undergo up-regulation, the excessive presence of CD8+ T cells, CD4+ T cells, and macrophages in cancer can tumor-induced immunosuppression, immune cell exhaustion, regulatory T cell activity, and the up-regulation of immune checkpoints and immunosuppressive molecules. These factors hinder the immune system’s ability to effectively combat cancer, underscoring the complexity of the tumor microenvironment [80,81]. Overall, this scenario implies that the up-regulation of MMP2, MMP9, MMP12, and MMP16 expression may contribute to KIRC aggressiveness and resistance to immunotherapy by altering the performance of CD8+ T, CD4+ T, and macrophages immune cells. Lastly, KEGG analysis further validated the connection of MMP2, MMP9, MMP12, and MMP16 hub genes with different cancer-driving pathways, such as “Bladder cancer, endocrine resistance, and relaxin signaling pathways.”

By integrating computational and molecular experiments, this study presents a comprehensive analysis of the MMP gene family, focusing on MMP2, MMP9, MMP12, and MMP16, in KIRC, shedding light on their potential as therapeutic targets and diagnostic biomarkers. Moreover, the identification of promoter hypomethylation as a regulatory mechanism linked to the up-regulation of hub genes adds an epigenetic dimension to the understanding of MMP gene regulation in KIRC.

While the study identifies promising therapeutic targets and diagnostic biomarkers in KIRC, it primarily relies on Bioinformatics and limited molecular analyses. Further functional experiments are needed to validate the specific roles of MMP2, MMP9, MMP12, and MMP16 in KIRC pathogenesis. The study does not explore the potential interplay between these hub genes and other factors influencing KIRC, such as microenvironmental factors. Clinical translation of these findings into targeted therapies and diagnostic assays would require additional validation and clinical trials.

## Conclusion

This study has uncovered valuable insights into the MMP gene family’s role within KIRC. Specifically, our findings highlight MMP2, MMP9, MMP12, and MMP16 as prominent molecular signatures and potential therapeutic targets in KIRC patients. These genes exhibit markedly elevated expression levels, coupled with aberrant promoter methylation patterns across KIRC samples. Furthermore, additional analyses revealed that the overexpression of these prominent MMP genes is associated with shorter OS, altered infiltration level of different immune cells, and dysregulation of various important pathways among KIRC patients. While these results are promising, it is imperative for future investigations to validate the oncogenic roles of these genes through rigorous biological experiments.

## Data Availability

The datasets analyzed in the current study can be found at http://ualcan.path.uab.edu/cgi-bin/ualcan-res-prot.pl.
